# Tumour grade significantly correlates with total dysfunction of tumour tissue-infiltrating lymphocytes in renal cell carcinoma

**DOI:** 10.1038/s41598-020-63060-1

**Published:** 2020-04-10

**Authors:** Atsunari Kawashima, Takayuki Kanazawa, Yujiro Kidani, Tetsuya Yoshida, Michinari Hirata, Kentaro Nishida, Satoshi Nojima, Yoshiyuki Yamamoto, Taigo Kato, Koji Hatano, Takeshi Ujike, Akira Nagahara, Kazutoshi Fujita, Akiko Morimoto-Okazawa, Kota Iwahori, Motohide Uemura, Ryoichi Imamura, Naganari Ohkura, Eiichi Morii, Shimon Sakaguchi, Hisashi Wada, Norio Nonomura

**Affiliations:** 1 Department of Urology, Graduate School of Medicine, Osaka University, Suita, Osaka Japan; 2 Department of Clinical Research in Tumour Immunology, Graduate School of Medicine, Osaka University, Suita, Osaka Japan; 3 Drug Discovery & Disease Research Laboratory, Shionogi & Co., Ltd., Toyonaka, Japan; 4 Department of Basic Research in Tumour Immunology, Graduate School of Medicine, Osaka University, Suita, Osaka Japan; 5 Department of Pathology, Graduate School of Medicine, Osaka University, Suita, Osaka Japan; 6 Department of Urological Immuno-Oncology, Graduate School of Medicine, Osaka University, Suita, Osaka Japan; 7 Department of Experimental Immunology, Immunology Frontier Research Centre, Osaka University, Suita, Osaka Japan

**Keywords:** Cancer microenvironment, Renal cell carcinoma

## Abstract

It is important to evaluate the clinical importance of both CD8 T cells and CD4 T cells expression simultaneously because they have crucial networks in tumour targeting immune responses. In 97 RCC patients, RNA sequencing and gene set enrichment analysis of both CD8 and CD4 T cells based on the expression levels of PD-1 and TIM-3 implied that the populations of PD-1+TIM-3+ CD8 T cells and PD-1lowTIM-3 + CD4 T cells were characterized as exhausted CD8 T cells and regulatory CD4 T cells, respectively. These populations of CD4 and CD8 T cells were significantly upregulated in the patients with RCC of higher WHO/ISUP grade (grades 3, 4) (*P* < 0.001). Moreover, the cytokine productivities of each population in both CD4 and CD8 T cells of the higher-grade patients were significantly lower than those of the lower-grade patients (*P* < 0.05). Multivariate analysis showed the prognosis of patients with metastatic RCC of higher WHO/ISUP grade treated by nivolumab to be significantly worse than that of patients with lower grade (*P* = 0.026). This study showed that tumour grade significantly correlated with dysfunction of both CD4+ and CD8+ TILs and the efficacy of nivolumab treatment.

## Introduction

Recently, cancer immunotherapy including monotherapy^1^ or combination therapy with anti-PD-1 antibody^2^ showed better therapeutic effect than molecular-targeted therapies for metastatic renal cell carcinoma (RCC) patients. However, the therapeutic effect of anti-PD-1 antibody monotherapy against RCC is limited, and both the establishment of biomarkers to predict efficacy and the development of new therapeutic target factors are necessary using tissue infiltrating lymphocytes (TILs).

Recently, subpopulations of CD4 and CD8 T cells stratified by PD-1 expression in the tumour microenvironment were reported to be important prognostic factors in cancer immunotherapy^3–5^. The transcriptomic data of TILs were published based not only on a single cell^6,7^ but also on the intensity of PD-1 expression^3^, and high expression of PD-1 was identified as a predictive marker in non-small cell lung cancer patients. Analysis of TILs according to the expression pattern of multiple immunocheckpoint (IC) molecules revealed the presence of terminally differentiated and stem like CD8 T cells in RCC patients^8^. However, few studies have simultaneously evaluated the functions of both CD8 T cells and CD4 T cells, which have crucial networks in establishing immune status in tumour microenvironments^9^ and tumour targeting immune responses^10–12^.

In this study, we performed RNA sequencing of both CD8 T cells and CD4 T cells based on the expression patterns of PD-1 and TIM-3 and aimed to clarify those characteristics and the differences stratified by tumour grade in RCC patients. In addition, we also evaluated the relationship between tumour grade and the efficacy of nivolumab monotherapy.

## Results

### Patient characteristics

Clinical characteristics of the 97 patients are listed in Supplementary Table 1. The histology of 86 of the patients (88.7%) was clear cell RCC. Twenty-nine patients (29.9%) had a higher World Health Organisation (WHO)/International Society of Urological Pathology (ISUP) grade of RCC (Grade 3 or 4).

Characteristics of the 20 patients treated with anti PD-1 antibody monotherapy are shown in Supplementary Table 2. Eleven patients (55.0%) had a higher WHO/ISUP grade, and seven patients (35.0%) were in the poor Memorial Sloan-Kettering Cancer Center (MSKCC) risk group. Most of the patients (70.0%) had received one or two prior therapies before nivolumab treatment, and none had been treated previously with IC inhibitors.

### Difference in expression patterns of PD-1 and TIM-3 by sample types among PBMCs, NILs, and TILs

**Figure 1:**
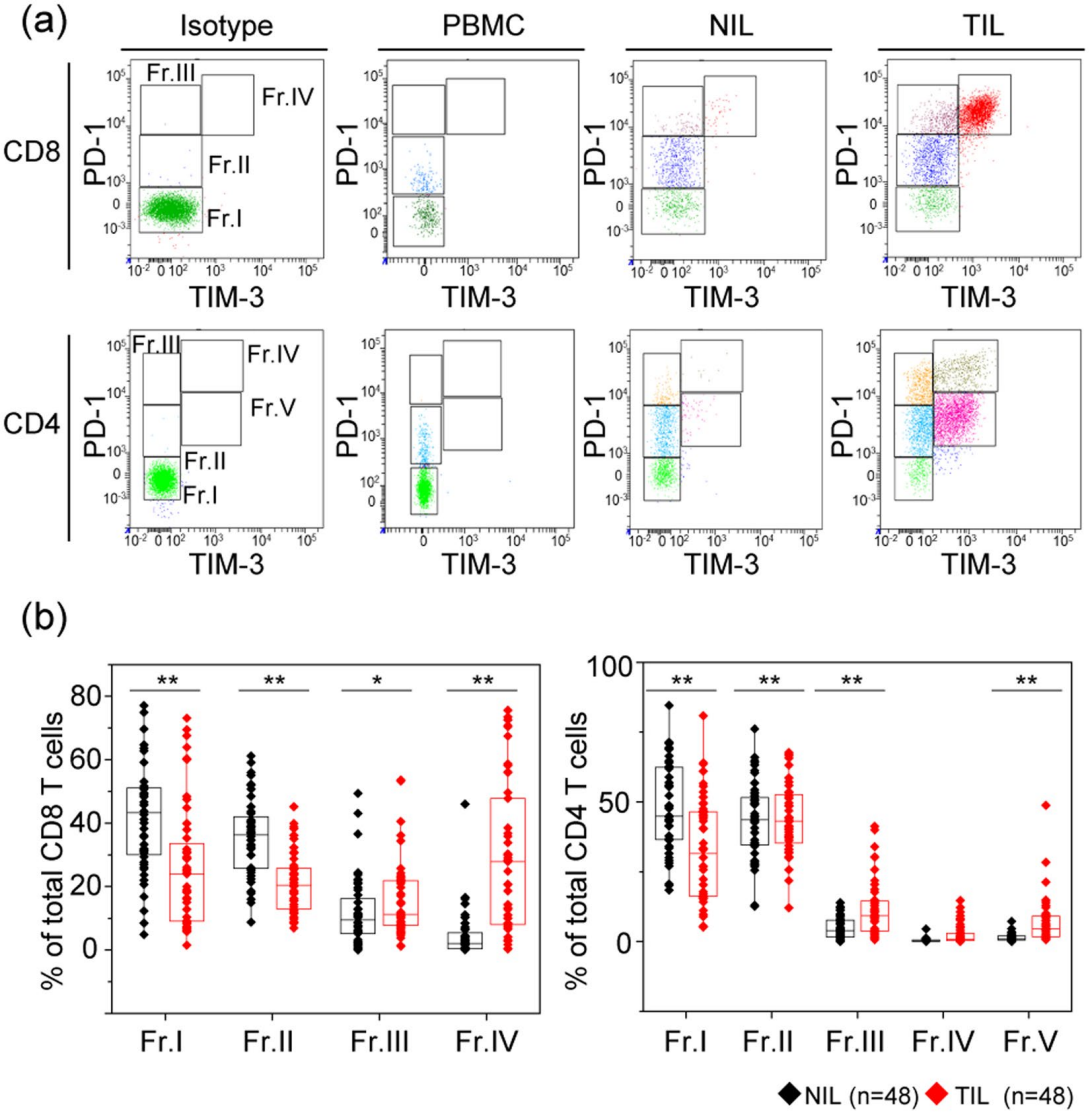
Co-expression pattern of PD-1 and TIM-3 on both CD8 and CD4 T cells of peripheral blood mononuclear cells (PBMC), adjacent normal tissue-infiltrating lymphocytes (NILs), and tumour tissue-infiltrating lymphocytes (TILs) in 48 patients with renal cell carcinoma. (**a**) Gating strategy to identify subsets of CD8 and CD4 T cells within PBMC, NILs, and TILs. CD8 T cells were classified into four groups (Fr. I: PD-1-TIM-3−, Fr. II: PD-1lowTIM-3−, Fr. III: PD-1+TIM-3−, Fr. IV: PD-1+TIM-3+), and CD4 T cells were classified into 5 groups (Fr. I: PD-1-TIM-3−, Fr. II: PD-1lowTIM-3−, Fr. III: PD-1+TIM-3−, Fr. IV: PD-1+TIM-3+, Fr. V: PD-1lowTIM-3+). (**b**) Expression ratios of each fraction on both CD4 and CD8 T cells were compared among NILs and TILs simultaneously extracted from the same patients. Comparison of two sample types was performed by Mann-Whitney U test. The central tendency of the box plot shows the median of each group, and the upper and lower ranges of the box plot show the 25^th^ and 75^th^ percentiles of each data set, respectively. **P < 0.01, *P < 0.05.

The representative expression patterns of PD-1 and TIM-3 on both CD4 T cells and CD8 T cells of peripheral blood mononuclear cells (PBMCs), adjacent normal tissue-infiltrating lymphocytes (NILs), and TILs are shown in Fig. 1a. In CD8 T cells, the intensity of PD-1 expression was highest in TILs, followed by NILs and PBMCs, and the expression of TIM-3 was found only with high expression of PD-1. Unlike other carcinomas, there are few cells with high TIM-3 and low PD-1 expression in RCC. In CD4 T cells, the intensity of PD-1 expression was highest in TILs, followed by NILs and PBMCs, and the populations with high TIM-3 expression were classified into two groups based on the intensity of PD-1 expression.

From the above results, CD8 T cells were fractionated into four groups (Fr. I: PD-1-TIM-3−, Fr. II: PD-1lowTIM-3−, Fr. III: PD-1highTIM-3−, and Fr. IV: PD-1highTIM-3+), and CD4 T cells were fractionated into 5 groups (Fr. I: PD-1-TIM-3−, Fr. II: PD-1lowTIM-3−, Fr. III: PD-1highTIM-3−, Fr. IV: PD-1highTIM-3+, and Fr. V: PD-1lowTIM-3+).

In 48 patients whose TILs and NILs were sampled simultaneously, the frequencies of NILs and TILs were significantly different in all fractions of CD8 T cells and all fractions but Fr. IV of CD4 T cells (Fig. 1b). Therefore, these 8 fractions showing a significant difference between NILs and TILs were examined in the subsequent analysis. In some patients, CD8+ CD103 + T cells, defined as tissue-resident memory T (Trem) cells^13^, were expressed in NILs and TILs (Supplementary Fig. 1).

### Significant difference in characteristics within CD8 TILs based on expression patterns of PD-1 and TIM-3

**Figure 2:**
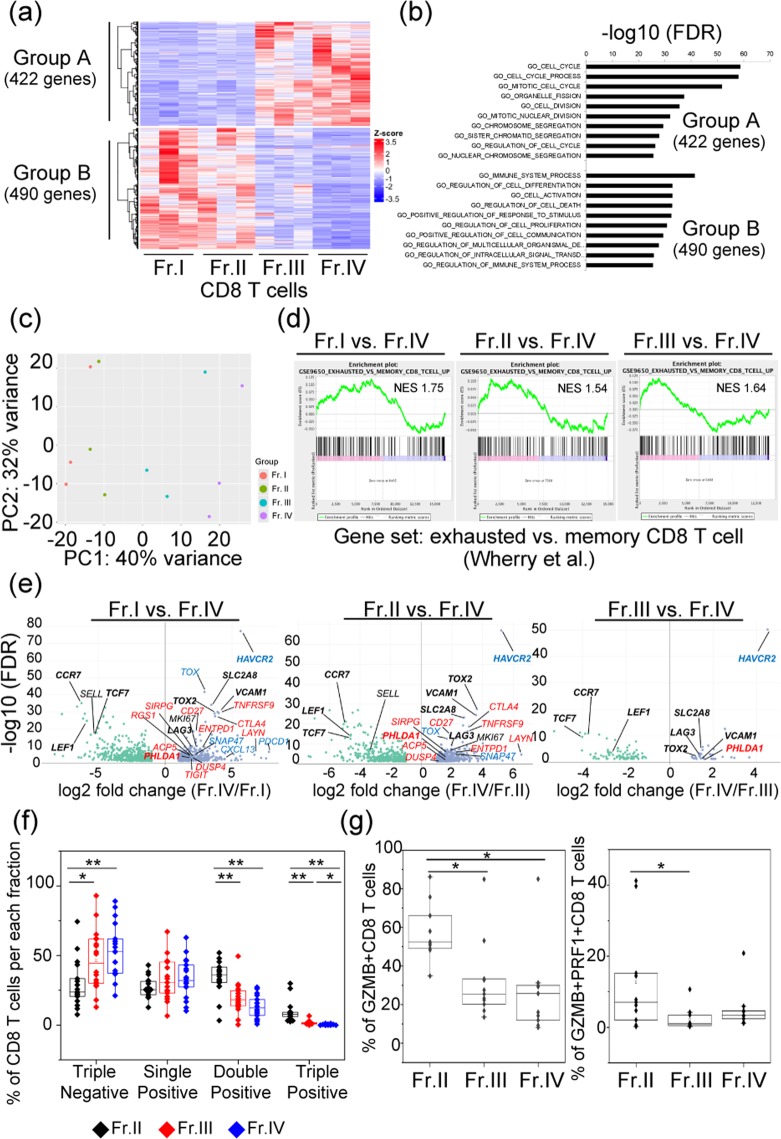
Gene expression profile and functional analysis of CD8 tumour tissue-infiltrating lymphocytes (TILs) based on the expression patterns of PD-1 and TIM-3. (**a**) Clustering analysis for genes distinguishing four subsets of CD8 TILs extracted from three patients with renal cell carcinoma. In total, 912 genes were significantly upregulated or downregulated (log_2_ fold change > [1], false discovery rate [FDR] < 0.05) as indicated by at least one group comparison. (**b**) Biological processes (Gene Ontology [GO] terms) enriched in group A and group B. (**c**) Principal component (PC) analysis of four subsets of CD8 T cells based on the expression patterns of PD-1 and TIM-3. (**d**) Gene set enrichment analysis (GSEA) of published data sets of biological processes (GO terms) enriched in group A and group B^14^. Statistical significance was determined by permutation testing with normalized enrichment score (NES). (**e**) Volcano plot of up- or downregulated genes between Fr. I vs. Fr. IV, Fr. II vs. Fr. IV, and Fr. III vs. Fr. IV. Genes specific only to exhausted T cells (blue) and to both exhausted CD8 T cells and CD4 regulatory T cells (Tregs) (red)^7^ are shown, and bold characters indicate the genes commonly having significant differences between these three combinations. (**f**) Comparison of multiple cytokine productivity (IFNγ, TNFα, and IL-2) of CD8 TILs from 18 patients in Fr. II, Fr. III, and Fr. IV among three sample types. (**g**) Cytotoxic functionality producing GZMB and both GZMB and PRF1 were compared within three fractions by Kruskal-Wallis analysis, and then a comparison of each sample type was performed by Bonferroni-corrected Mann-Whitney U test. The central tendency of the box plot indicates the median of each group, and the upper and lower ranges of the box plot show the 25^th^ and 75^th^ percentiles of each data set, respectively. **P < 0.01, *P < 0.05.

Transcriptomic analysis of each fraction performed by RNA sequencing using 12 fractions and hierarchical clustering analysis using 912 genes mainly revealed two clusters composed of two groups (group A: Fr. I/Fr. II and group B: Fr. III/Fr. IV) (Fig. 2a). Pathway enrichment analysis showed that 422 genes of group A were involved in cell cycle regulation and proliferation and were already reported to correlate with high expression of PD-1 in TILs in lung cancer^3^, and 490 genes of group B were involved in immune system processes (Fig. 2b). Principal component analysis (PCA) of these genes revealed that all samples of Fr. I and Fr. II were located nearby each other, and all samples of Fr. III and Fr. IV were located closer to, rather than farther from, each other (Fig. 2c). Gene set enrichment analysis (GSEA) of published data sets from CD8 T cell exhaustion during chronic viral infection^14^ was performed for each fraction, and the exhaustion-related genes were significantly enriched in Fr. IV compared with those in the other fractions (Fig. 2d).

Compared with Fr. I and Fr. II, suppressive immune-related genes such as *LAG3*, *CTLA4*, and *HAVCR2* were significantly upregulated in Fr. IV (Fig. 2e). Especially, among 21 genes specific to exhausted T cells identified by single-cell RNA sequencing^7^, 16 genes (76.2%) were upregulated in Fr. IV compared with 13 genes (61.9%) upregulated in Fr. I and Fr. II. Also 9 of 12 genes reported to be commonly elevated in both exhausted CD8 T cells and CD4 regulatory T cells (Tregs)^7^ were upregulated in Fr. IV compared to both Fr. I and Fr. II. Only expression levels of *HAVCR2* and *PHLDA1* among the above 21 genes were upregulated when comparing Fr. III and Fr. IV. Fourteen genes including *LAG3* and *TOX2* were consistently upregulated in Fr. IV compared to Frs. I, II, and III. Moreover, genes such as *TCF7*, *CCR7*, and *LEF1* specific to naïve CD8 T cells identified by single-cell RNA sequencing^7^ were consistently downregulated in Fr. IV compared to Frs. I, II, and III (Fig. 2e).

TCR clonal analysis showed that diversities of T-cell receptor (TCR) clonotypes of both α chain (TCRA) and β chain (TCRB) were the lowest in Fr. IV among the 4 populations although the difference was not significant (Supplementary Fig. 2a,b). Clonotypes with specific proportions tended to be higher in Fr. IV among 3 patients (Supplementary Fig. 2c), and some clonotypes of TCRA and TCRB were shared among the 4 fractions within each individual (Supplementary Fig. 2d). There were no differences in the proportion of clonotypes according to the expression of CD103 as a feature of Trem cells (Supplementary Figs. 1 and 2c).

The expression ratio of CD8 T cells producing all three cytokines (IL-2, TNFα, and IFNγ) in Fr. IV was significantly lower than that of Fr. II and Fr. III (Fig. 2f). In addition, the expression ratio of CD8 T cells producing either of two kinds of cytokines was significantly higher in Fr. II compared to Fr. III and Fr. IV, and the expression ratio of CD8 T cells producing no cytokines was higher in Fr. III and Fr. IV compared to Fr. II.

In terms of cytotoxic functionality, the expression ratio of CD8 T cells producing GZMB was significantly higher in Fr. II compared to Fr. III and Fr. IV (*P* = 0.018), and that of CD8 T cells producing both GZMB and PRF1 was higher in Fr. II compared to Fr. III and Fr. IV in 11 patients (*P* = 0.013) (Fig. 2g).

The above results showed that depending on the expression of PD-1 and TIM-3, the lineage of CD8 T cells correlated significantly with the dysfunction of these cells. Especially, CD8+ PD-1highTIM-3+ was the most functionally impaired population within the four groups.

### Significant difference in characteristics within CD4 TILs based on expression patterns of PD-1 and TIM-3

**Figure 3:**
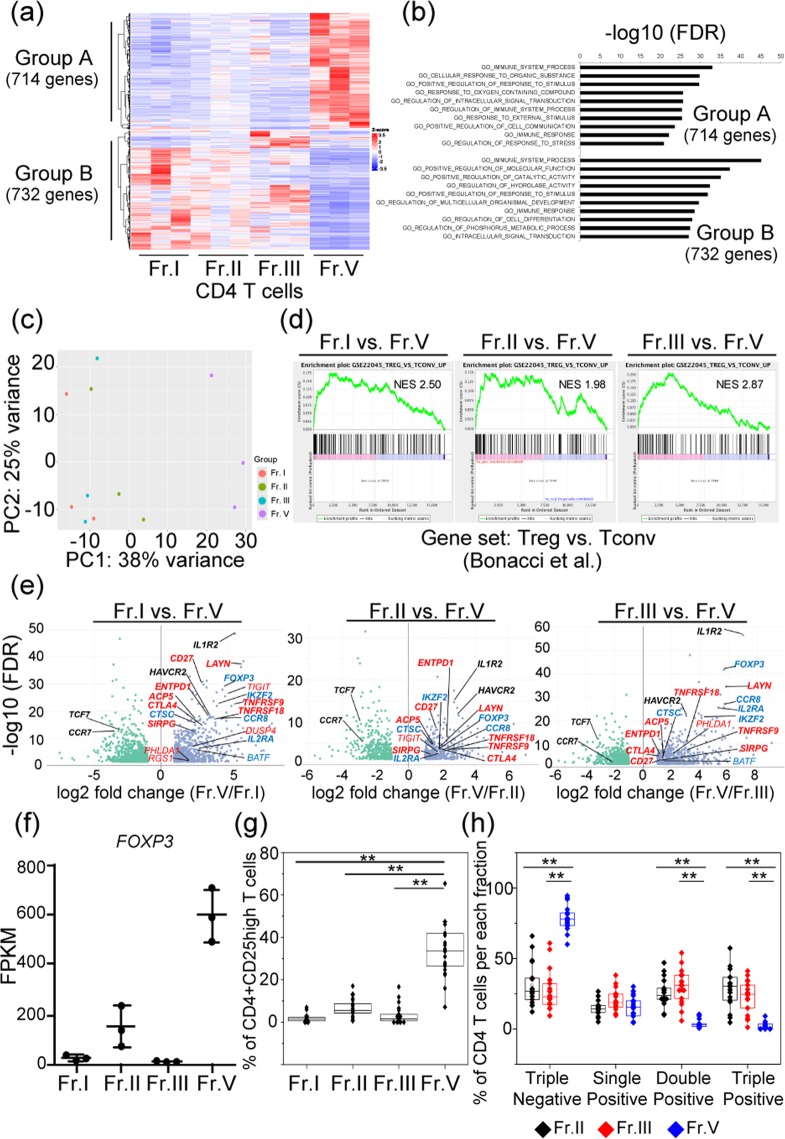
Gene expression profile and functional analysis of CD4 tumour tissue-infiltrating lymphocytes (TILs) based on the expression patterns of PD-1 and TIM-3. (**a**) Clustering analysis for genes distinguishing four subsets except for Fr. IV within five fractions of CD4 TILs extracted from three patients with renal cell carcinoma. In total, 1446 genes were significantly upregulated or downregulated (log_2_ fold change > [1], false discovery rate [FDR] < 0.05) as indicated by at least one group comparison. (**b**) Biological processes (Gene Ontology [GO] terms) enriched in group A and group B. (**c**) Principal component (PC) analysis for four subsets of CD4 T cells based on the expression patterns of PD-1 and TIM-3. (**d**) Gene set enrichment analysis (GSEA) of published data sets of biological processes (GO terms) enriched in group A and group B^17^. Statistical significance was determined by permutation testing with normalized enrichment score (NES). (**e**) Volcano plot of up- or downregulated genes between Fr. I vs. Fr. IV, Fr. II vs. Fr. IV, and Fr. III vs. Fr. IV. Genes specific only to CD4 regulatory T cells (Tregs) (blue) and to both exhausted CD8 T cells and Tregs (red)^7^ are shown, and bold characters indicate the genes commonly having significant differences between these three combinations. (**f**) Gene expression of *FOXP3* among each fraction. (**g**) Comparison of the expressions of CD4 + CD25high T cells, previously defined as CD4 Tregs, is stratified by each fraction. (**h**) Comparison of multiple cytokine productivity (IFNγ, TNFα, and IL-2) of CD4 TILs from 18 patients in Fr. II, Fr. III, and Fr. V. among three sample types was performed by Kruskal-Wallis analysis, and then a comparison of each sample type was performed by Bonferroni-corrected Mann-Whitney U test. The central tendency of the box plot indicates the median of each group, and the upper and lower ranges of the box plot show the 25^th^ and 75^th^ percentiles of each data set, respectively. **P < 0.01.

As with the CD8 T cells, transcriptomic analysis of each fraction was performed by RNA sequencing using 12 fractions, except for three fractions of Fr. IV, and hierarchical clustering analysis using 1446 genes mainly revealed two clusters that were classified into two groups (group A: Fr. I/Fr. II/Fr. III and group B: Fr. V) (Fig. 3a). Pathway enrichment analysis revealed 714 genes of group A that were involved in immune responses and were already reported to correlate with CD4 Tregs^15,16^ (Fig. 3b). PCA of these genes revealed that three samples of Fr. V were located apart from the other 9 samples of Fr. I/Fr. II and Fr. III (Fig. 3c). GSEA of published gene data sets specific to CD4 Tregs^17^ was performed among each fraction, and the Treg-specific genes were significantly enriched in Fr. V compared to Frs. I, II, and III (Fig. 3d).

Compared with that in Frs. I, II, and III, *FOXP3* was significantly upregulated in Fr. V along with 13 of 20 genes characterized as CD4 Treg-specific genes^7^ (Fig. 3e,f). Also 8 of 12 genes reported to be commonly elevated in both exhausted CD8 T cells and CD4 Tregs^7^ were upregulated in Fr. V compared that in to Frs. I, II, and III. Moreover, genes such as *TCF7* and *CCR7*, which were enriched in naïve CD4 T cells identified by single-cell RNA sequencing^7^, were consistently downregulated in Fr. V compared to that in Frs. I, II, and III.

These results indicated that CD4 Tregs were enriched in the Fr. V population. Actually, the population of CD4 + CD25high T cells in Fr. V, which were reported to be the major population of CD4 Tregs in RCC^18^, was significantly higher compared to those in Fr. I/Fr. II and Fr. III (Fig. 3g).

TCR clonal analysis showed that diversities of TCR clonotypes of both TCRA and TCRB were the lowest in Fr. III and Fr. V among the 4 populations although the difference was not significant (Supplementary Fig. 3a,b). Clonotypes with specific proportions tended to be higher in Fr. III of 2 patients (Supplementary Fig. 3c), and none of the clonotypes of TCRA were shared among all 4 fractions although some clonotypes of TCRA and TCRB were shared among Fr. I, Fr. II, and Fr. III within each individual (Supplementary Fig. 3d).

The expression ratio of CD4 T cells producing all three cytokines (IL-2, TNFα, and IFNγ) in Fr. V was significantly lower than that of Fr. II and Fr. III (Fig. 3h). In addition, the expression ratio of CD4 T cells producing either of two kinds of cytokines was significantly higher in Fr. II and Fr. III compared to Fr. V, and the expression ratio of CD4 T cells producing no cytokines was higher in Fr. V compared to Fr. II and Fr. III (Fig. 3h).

The above results showed that one specific population of CD4 T cells (CD4 + PD-1lowTIM-3+) correlated significantly with dysfunction of CD4 T cells that was similar to that in CD4 Tregs.

### Significant correlation of tumour grade with dysfunctional populations in both CD4 and CD8 T cells

**Figure 4:**
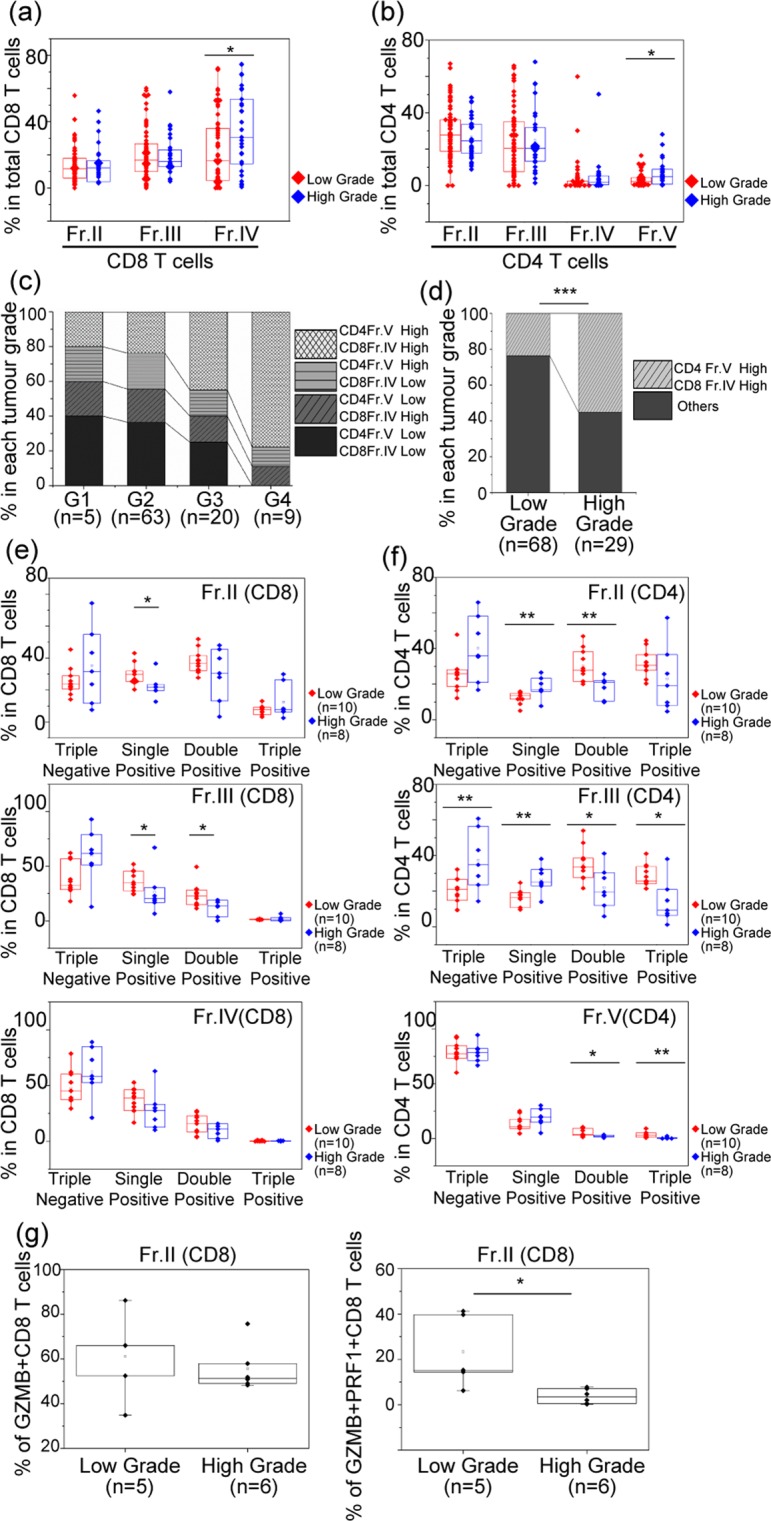
The relationship between total dysfunction of CD8 and CD4 T cells and tumour grade. (**a**) Comparison of the expression ratio of each fraction of CD8 T cells stratified by tumour grade. (**b**) Comparison of the expression ratio of each fraction of CD4 T cells stratified by tumour grade. For this panel, the comparison was performed by Mann-Whitney U test. The central tendency of the box plot shows the median of each group, and the upper and lower ranges of the box plot show the 25^th^ and 75^th^ percentiles of each data set, respectively. *P < 0.05. (**c**) The ratio of the patients stratified by expression pattern of CD4 T cells and CD8 T cells with dysfunctional populations according to tumour grade. Dysfunctional populations were divided into two groups based on the median expression level (Fr. IV of CD8 T cells: 20.3%, Fr. V of CD4 T cells: 2.79%). (**d**) The ratio of the patients with high expression ratio of both CD4 T cells and CD8 T cells with dysfunctional populations according to tumour grade. ***P < 0.001. (**e**) Comparison of multiple cytokine productivity (IFNγ, TNFα, and IL-2) of CD8 TILs from 18 patients stratified by tumour grade within Fr. II, Fr. III, and Fr. V. (**f**) Comparison of multiple cytokine productivity (IFNγ, TNFα, and IL-2) of CD4 TILs from 18 patients stratified by tumour grade within Fr. II, Fr. III, and Fr. IV. **P < 0.01, *P < 0.05. (**g**) Cytotoxic functionality producing GZMB and both GZMB and PRF1 in Fr. II of CD8 T cells is compared according to tumour grade. Comparison of two sample types was performed by Mann-Whitney U test. The central tendency of the box plot indicates the median of each group, and the upper and lower ranges of the box plot show the 25^th^ and 75^th^ percentiles of each data set, respectively. *P < 0.05.

Previously, we reported that immunological classification based on IC molecules was significantly correlated with tumour grade in RCC patients^18^. Stratified by tumour grade, the expression ratio of Fr. IV in CD8 T cells and Fr. V in CD4 T cells, which were both defined as dysfunctional populations in each T cell, were significantly upregulated in the patients with higher tumour grade (WHO/ISUP grades 3 and 4) (n = 29) rather than lower tumour grade (WHO/ISUP grades 1 and 2) (n = 68) (Fr. IV in CD8 T cells: *P* = 0.030, Fr. V in CD4 T cells: *P* = 0.029) (Fig. 4a,b).

When these dysfunctional populations were divided into two groups based on the median expression level (Fr. IV of CD8 T cells: 20.3%, Fr. V of CD4 T cells: 2.79%), 20% (1/5) of the patients with WHO/ISUP grade 1 and 23.8% (15/63) with WHO/ISUP grade 2 had higher expressions of both populations. In contrast, 45% (9/20) of the patients with WHO/ISUP grade 3 and 77.7% (7/9) with WHO/ISUP grade 4 had higher expressions of both populations (Fig. 4c). Overall, the ratio of patients with higher dysfunctional populations of both CD4 T and CD8 T cells was significantly higher in those with higher (16/29, 55.2%) rather than lower tumour grade (16/58, 23.5%) (*P* = 0.004) (Fig. 4d). In multivariate analysis along with other clinical items including tumour stage, only tumour grade significantly correlated with higher dysfunctional populations of both CD4 T and CD8 T cells (odds ratio: 3.48, 95% confidence interval [CI]: 1.39–8.73, *P* = 0.008) (Table 1).

**Table 1:** Univariate and multivariate analysis of logistic regression of clinical characteristics correlated with high expression of both CD8 and CD4 dysfunctional populations.

	Univariate analysis			Multivariate analysis		
	OR	95% CI	*P*	OR	95% CI	*P*
Age (<66 vs. ≥66 years)	0.39	0.08–1.88	0.239			
Gender (Male vs. Female)	1.72	0.68–4.34	0.252			
WHO/ISUP grade (1, 2 vs. 3, 4)	**3.48**	**1.39–8.73**	**0.008**	**3.48**	**1.39–8.73**	**0.008**
Clinical T stage (I, II vs. III, IV)	1.66	0.69–4.03	0.261			
Coagulative necrosis (absence vs. presence)	1.62	0.63–4.72	0.319			
Histological type (clear cell vs. non clear cell)	0.50	0.10–2.51	0.399			
Lymphovascular invasion (No vs. Yes)	2.37	0.97–5.82	0.059			
NLR (<3.45 vs. ≥3.45)	**3.17**	**1.31–7.68**	**0.011**			0.106
PLR (<1.61 vs. ≥1.61)	**3.33**	**1.35–8.21**	**0.009**			0.121
CRP (<1 mg/dL vs. ≥1 mg/dL)	2.29	0.82–6.40	0.114			

Abbreviations: OR = odds ratio, CI = confidence interval, NLR = neutrophil to lymphocyte ratio, PLR = platelet to lymphocyte ratio, CRP = C reactive protein.

### Cytokine productivities of both CD4 and CD8 T cells decreased significantly in the patients with higher versus lower tumour grade

Interestingly, we noticed heterogeneity in the results of the ability to produce cytokines even within the same fraction (Figs. 2f and 3h). The results of the multi-functionality assay stratified by tumour grade showed that in CD8 T cells, the abilities to produce a single cytokine in Fr. II and Fr. III, and two kinds of cytokines in Fr. III, were significantly higher in the patients with lower (n = 11) rather than higher tumour grade (n = 7) (*P* < 0.05) (Fig. 4e). In contrast, there was no significant difference in cytokine productivity depending on tumour grade in Fr. IV of CD8 T cells. However, in the CD4 T cells, the abilities to produce two kinds of cytokines in Frs. II, III, and V and three kinds of cytokines in Frs. III and V were significantly greater in the patients with lower rather than higher tumour grade (*P* < 0.05) (Fig. 4f).

Within 12 sets of TILs from the patients with higher dysfunctional populations of both CD4 T and CD8 T cells, cytokine productivities of only CD4 T cells (two kinds of cytokines in Fr. II [*P* = 0.030] and three kinds of cytokines in Fr. III [*P* = 0.030] and Fr. V [*P* = 0.048]) were significantly greater in the patients with lower rather than higher tumour grade (Supplementary Fig. 4).

With regard to cytotoxic functionality, there was no significant difference in the expression ratio of Fr. II of CD8 T cells producing granzyme B (GZMB) depending on the tumour grade. However, the expression ratio of Fr. II of CD8 T cells producing both GZMB and perforin (PRF1) was greater in the patients with lower rather than higher tumour grade (*P* = 0.017) (Fig. 4g). Within 9 sets of TILs from the patients with higher dysfunctional populations of both CD4 T and CD8 T cells, there was no significant difference of cytotoxic functionality between lower and higher tumour grade.

These results suggested that tumor grade correlated not only with the induction of dysfunctional populations in CD8 T cells and CD4 Tregs but also with the decline of cytokine productivities of both CD8 and CD4 TILs.

### Significant correlation of tumour grade with prognosis of RCC patients treated with nivolumab monotherapy

Among the 97 patients, 10 were treated with nivolumab monotherapy as second-line or higher treatments (Supplementary Fig. 5a). Five patients (50%) had higher expression of both Fr. IV of CD8 T cells and Fr. V of CD4 T cells, and 6 patients had higher tumour grade. Tumour grade could have stratified the prognosis of cancer-specific survival (CSS) better than the expression of Fr. IV of CD8 T cells and Fr. V of CD4 T cells (Supplementary Fig. 5b).

**Figure 5:**
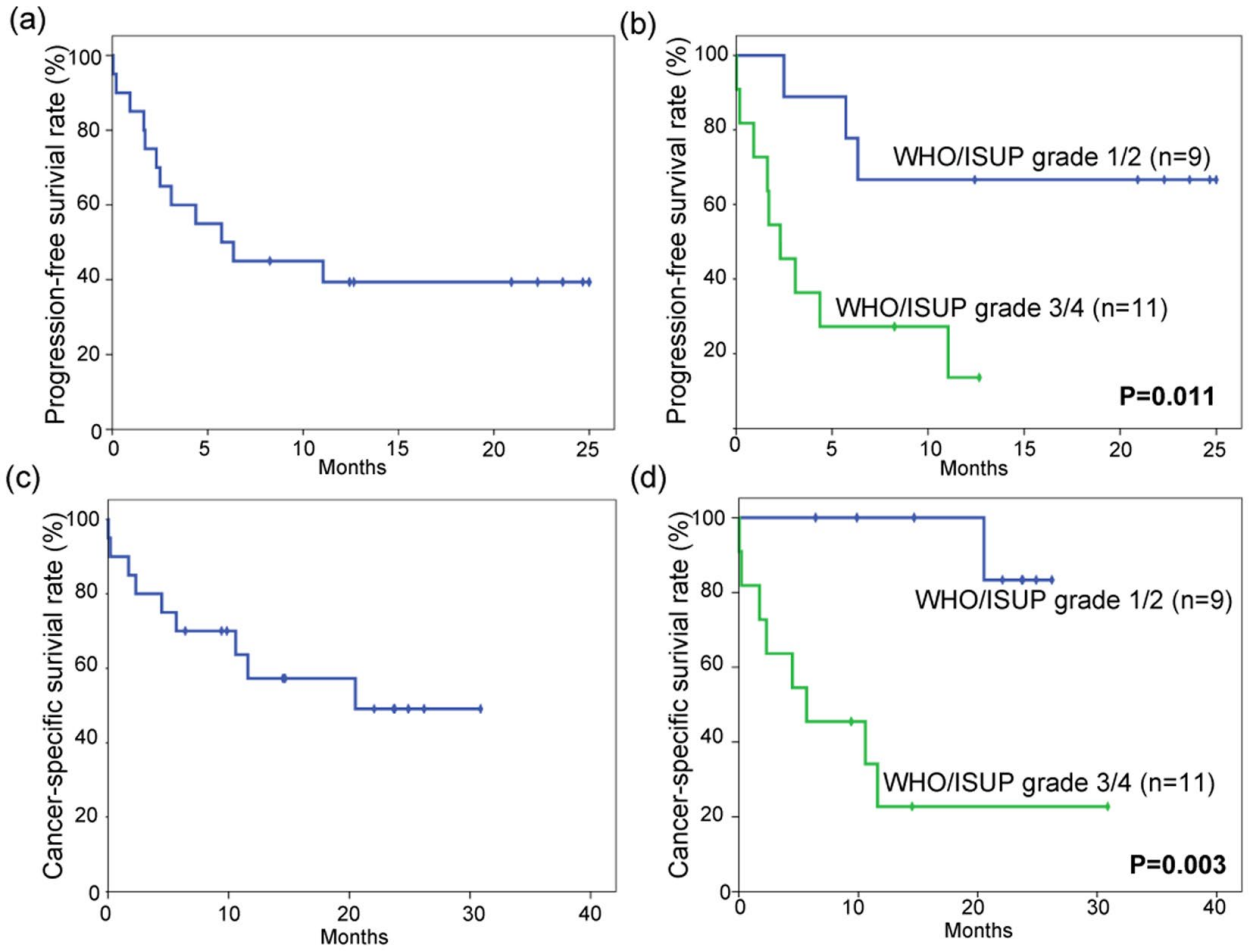
Probability estimates of progression-free survival time of patients with metastatic renal cell carcinoma treated with nivolumab (**a**) (n = 20) and stratification by tumour grade (**b**). Also shown are the probability estimates of cancer-specific survival time of the same patients (**c**) (n = 20) and stratification by tumour grade (**d**). Statistical analysis was performed by log-rank test.

Finally, 20 RCC patients treated with nivolumab monotherapy as second-line or higher treatments were evaluated for therapeutic effect stratified by tumour grade (Supplementary Table 2). The median progression-free survival (PFS) of all patients was 6.35 months (Fig. 5a). In the subgroup analyses, PFS of the patients with lower-grade tumours (median PFS: not reached, n = 9) was better than that of patients with higher-grade tumours (median PFS: 2.3 months, n = 11) (*P* = 0.011) (Fig. 5b).

The median CSS of all patients was 20.5 months (Fig. 5c). Subgroup analyses showed CSS of the patients with lower-grade tumours (median CSS: not reached, n = 9) to be better than that of patients with higher-grade tumours (n = 11) (median CSS: 5.66 months, *P* = 0.003) (Fig. 5d).

Multivariate Cox regression analysis identified higher tumour grade (hazard ratio [HR]: 4.63, 95% CI: 1.10–19.45, *P* = 0.036) to be an independent predictor of inferior PFS time along with MSKCC risk classification (*P* = 0.002) (Table 2). Similarly, higher tumour grade was also identified as an independent predictor of CSS time (HR: 22.49, 95% CI: 1.44–350.42, *P* = 0.026) along with MSKCC risk classification (*P* = 0.006) (Table 3).

**Table 2:** Univariate and multivariate analysis of prognostic factors for progression-free survival of metastatic RCC patients treated by nivolumab monotherapy.

	Univariate analysis			Multivariate analysis		
	HR	95% CI	*P*	HR	95% CI	*P*
Age (<66 vs. ≥66 years)	1.08	0.35–3.35	0.901			
Gender (Male vs. Female)	2.18	0.64–7.50	0.216			
WHO/ISUP grade (1, 2 vs. 3, 4)	**4.80**	**1.27–18.10**	**0.020**	**4.63**	**1.10–19.45**	**0.036**
Coagulative necrosis (absence vs. presence)	1.39	0.44–4.42	0.576			
MSKCC risk classification (Good/Intermediate vs. Poor)	**8.83**	**2.47–31.60**	**0.001**	**9.05**	**2.22–36.86**	**0.002**
Number of prior therapies (1 vs. ≥2)	0.66	0.21–2.08	0.479			
NLR (<3.45 vs. ≥3.45)	2.98	0.89–10.03	0.078			
PLR (<1.61 vs. ≥1.61)	2.27	0.68–7.59	0.185			
CRP (<1 mg/dL vs. ≥1 mg/dL)	2.69	0.80–9.00	0.109			

Abbreviations: RCC = renal cell carcinoma, HR = hazard ratio, CI = confidence interval, MSKCC = Memorial Sloan Kettering Cancer Center, NLR = neutrophil to lymphocyte ratio, PLR = platelet to lymphocyte ratio, CRP = C reactive protein.

**Table 3:** Univariate and multivariate analysis of prognostic factors for cancer-specific survival of metastatic RCC patients treated by nivolumab monotherapy.

	Univariate analysis			Multivariate analysis		
	HR	95% CI	*P*	HR	95% CI	*P*
Age (<66 vs. ≥66 years)	1.33	0.36–4.99	0.670			
Gender (Male vs. Female)	1.98	0.49–8.07	0.341			
WHO/ISUP grade (1, 2 vs. 3, 4)	**13.9**	**1.64–117.4**	**0.016**	**22.49**	**1.44–350.4**	**0.026**
Coagulative necrosis (absence vs. presence)	1.39	0.37–5.27	0.630			
MSKCC risk classification (Good/Intermediate vs. Poor)	**14.8**	**2.97–73.66**	**0.001**	**21.14**	**2.41–185.2**	**0.006**
Number of prior therapies (1 vs. ≥2)	0.52	0.14–1.98	0.338			
NLR (<3.45 vs. ≥3.45)	3.05	0.75–12.36	0.119			
PLR (<1.61 vs. ≥1.61)	1.88	0.47–7.52	0.374			
CRP (<1 mg/dL vs. ≥1 mg/dL)	2.79	0.68–11.41	0.153			

Abbreviations: RCC = renal cell carcinoma, HR = hazard ratio, CI = confidence interval, MSKCC = Memorial Sloan Kettering Cancer Center, NLR = neutrophil to lymphocyte ratio, PLR = platelet to lymphocyte ratio, CRP = C reactive protein.

## Discussion

The evolution of cancer immunotherapy using anti-PD-1 antibody has been remarkable for various cancer types including RCC^1,2^. Several combination therapies targeting PD-1^2,19,20^ showed better therapeutic effect compared to conventional molecular-targeted therapies, and the first combination was approved as first-line therapy for patients with metastatic or advanced RCC^21^. However, no predictive or prognostic biomarkers including PD-L1 expression were established in these randomized phase III studies in RCC^1,2^, unlike the other cancers^22–24^. One reason might be that compared with other cancer types^25^, the number of TILs was originally a poor prognostic factor in RCC, and PD-L1 expression was a significant prognostic factor in RCC patients^26^. Therefore, other types of markers focusing on the functions of TILs should be identified in RCC.

This is the first study, to our knowledge, to show transcriptome data using RNA-Seq based on TIM-3 expression in RCC patients with high PD-1 expression although there was a recently published study on transcriptome data based on the intensity of PD-1 in non-small lung cancer patients^3^. In CD8 T cells, the population of CD8+ PD-1highTIM-3+ had the lowest function among the four groups analysed, and this population was significantly upregulated in the patients with higher rather than lower tumour grade as previously reported^9,27^. In the present study, there was only slight functional difference between the presence and absence of TIM-3 expression on CD8 T cells highly expressing PD-1. However, both TOX and TOX2, which were recently reported to impose CD8+ T cell exhaustion^5^, were significantly upregulated in CD8+ PD-1highTim-3+ compared to the other populations. So, upregulation of CD8+ PD-1highTim-3+ in the tumour microenvironment could potentially become an important predictive factor in cancer immunotherapy. In CD4 T cells, the population of CD4 + PD-1lowTIM-3+ had the lowest function among the four groups analysed. In previously published data on the immune landscape in RCC, there was one distinct population of CD4 T cells that showed mid-level expression of PD-1/TIM-3 and high-level expression of FOXP3. This population was defined as Tregs and was upregulated at an advanced stage compared to an early stage, which agreed with our data^9^. Recently, PD-1 + CD4 Tregs in the tumour microenvironment were suggested to induce hyper-progressive disease after anti PD-1 monotherapy^4^. So, upregulation of CD4 + PD-1lowTIM-3+ in the tumour microenvironment also could potentially become an important predictive factor along with CD8+ PD-1highTIM-3+ cells.

Interestingly, the present study showed the presence of functional heterogeneity even in the same phenotypic populations of both CD4 T cells and CD8 T cells, and it was dependent on tumour grade. Especially, the functional differences between the lower- and higher-grade tumours were remarkable in the CD8+ PD-1highTIM-3− cells and CD4 + PD-1highTIM-3− cells. In CD8+ T cells, CD8+ PD-1+TIM-3− cells were reported to contain stem-like CD8 T cells with higher proliferative potential. This study supposed that stem-like CD8 T cells might be more abundant in the lower-grade tumours though we couldn’t evaluate in this study^8^. In CD4 T cells, Yang *et al*. reported the presence of heterogeneity of PD-1-expressing CD4 T cells and identified a functionally antithetical population within CD4 T cells expressing PD-1 in follicular lymphoma^28^. In RCC patients, clinically important populations of CD4 T cells, except for Tregs, could not be identified in previous reports in which analysis was performed using 19 kinds of markers^9^. It has been suggested that CD4 T cells might play an important role as well as CD8 T cells in immunotherapy using IC inhibitors^10–12^ and further analysis is needed to identify the important population in CD4 T cells.

This study was also the first, to our knowledge, to show that WHO/ISUP grade could be a significant prognostic factor in RCC patients treated with anti-PD-1 antibody. Among RCC patients treated with anti PD-1 antibody monotherapy, the prognosis of the patients with a high PD-L1 expression was poorer than that in the patients with low PD-L1 expression^1^. Contrastingly, among RCC patients treated with combination therapy of anti PD-1 antibody and anti CTLA-4 antibody, the prognosis of the patients with high PD-L1 expression was better than that in the patients with low PD-L1 expression^2^. Moreover, the treatment efficacy of this combination was significantly superior to that of sunitinib in 112 patients with sarcomatoid tumour categorized as WHO/ISUP grade 4 regardless of PD-L1 expression according to the result of the combination of nivolumab plus ipilimumab in RCC patients (CheckMate 214)^29^. Previously, specific gene profiles correlating with sarcomatoid differentiation and T-cell infiltration were reported to predict the prognosis of metastatic RCC patients treated with sunitinib^30^. Interestingly, those genes included *CXCL13* and *PDCD1*, which were upregulated in Fr. III and Fr. IV of CD8 T cells compared to Fr. I in the present study. So, the combination therapy of ipilimumab and nivolumab targeting both CD4 + PD-1lowTIM-3+ and CD8+ PD-1highTIM-3+ might be able to reverse the natural prognosis of RCC and be ideal for treating patients with higher tumour grade. Actually, this novel immune therapy was effective in metastatic RCC patients with intermediate and poor risk, but the superiority of nivolumab and ipilimumab could not be shown in metastatic RCC patients with good risk^2^. We speculated that one reason might be the presence of more patients with lower-grade RCC among the metastatic RCC patients with good risk, and combination therapy was unnecessary for these patients, although we could not verify the results of the clinical trial. The WHO/ISUP grading system is widely recognized to be useful for the prognostic prediction of clear cell and papillary RCC and is usually used to evaluate only these two histological types^31^. However, this system could be used to describe the morphological features of all histological types of RCC, and thus we should consider tumour grade across tumour subtypes when evaluating the efficacy of IC inhibitor treatments because it is more versatile than the evaluation of immunohistochemistry in general practice. In our cohort, only surgical specimens were used to define WHO/ISUP grade because the diagnostic accuracy of tumour grade between biopsy and surgical pathology was reported to be around 50–75%^32,33^. Recently, the importance and versatility of biopsy of the renal mass or metastatic lesions have rapidly increased, and the development of a means of accurate diagnosis using liquid biopsy is needed^34^.

This study has several limitations. First, we could not clarify a direct association between total dysfunction of TILs and tumour grade because our extraction method was difficult to analyse alive tumour cells, especially in RCC tissues. Second, this was a retrospective study, and the number of patients sampled and treated with nivolumab monotherapy was small. Moreover, the sample types were mostly extracted from untreated primary lesions. The lymphocyte subsets between primary and metastatic RCC are reported to be slightly different^18^, and thus we need to perform an expanded study using liquid biopsy to represent the total condition of the tumour.

In conclusion, this study showed that tumour grade correlated significantly with the overall dysfunction of CD4 and CD8 T cells and the efficacy of nivolumab monotherapy. These results suggested that we should revisit the importance of cell morphology along with genetic features because cancer has heterogeneous features that are difficult to overcome^35^. Also, ipilimumab in combination with nivolumab might be preferable to nivolumab alone in patients with high-grade tumour after second-line therapy for RCC although there is no supporting evidence for this suggestion at present.

## Materials and Methods

### Patient characteristics

Two independent cohorts were analysed for this study as shown in Supplementary Fig. 1. Ninety-seven tumour tissues and 48 adjacent normal tissues were collected from 97 RCC patients surgically treated from October 2014 to May 2017 without perioperative multidisciplinary therapies (Supplementary Fig. 6a). The initially diagnosed tumours were staged according to the 7^th^ American Joint Committee on Cancer staging classification^36^. Pathological diagnosis was evaluated according to WHO/ISUP classification, grading, presence of coagulative necrosis and pathological staging of RCC^31^ by two specialized pathologists (N.S., M.E.). Patient characteristics including laboratory findings were evaluated as previously reported^18^.

Twenty patients treated with nivolumab monotherapy from November 2016 to December 2018 were retrospectively evaluated to clarify the relationship between tumour grade and their prognosis. Patients were evaluated at the time of nivolumab administration according to MSKCC risk groups for previously treated patients^37^, and tumour grade was diagnosed using surgical specimens, not biopsy specimens, obtained from primary or metastatic sites (Supplementary Fig. 6b).

This study was approved by the local ethics committee of the Graduate School of Medicine, Osaka University (approval nos. #708, #13397–2, #14069-3), and written informed consent was obtained from all patients. All experiments were performed in accordance with relevant guidelines and regulations.

### Preparation of lymphocytes analysed by multicolour flow cytometry

PBMCs were extracted from 1 mL peripheral blood of RCC patients using BD Pharm Lyse lysing buffer (BD Biosciences, Tokyo, Japan). Ninety-seven TILs were extracted macroscopically from cancer tissues, and 48 NILs were extracted macroscopically from non-cancerous tissues just after surgery and all fresh samples were analysed by flowcytometry and RNA sequencing. In several cases where coagulative necrosis was difficult to distinguish macroscopically, extracted area was evaluated later by haematoxylin and eosin staining. Details of lymphocyte extraction, methods, and clones of the antibodies for staining cell surface molecules were as previously reported^18^.

### T-cell sorting and RNA sequencing

Stained fresh T cells were sorted by FACSAria SOAP (BD Biosciences) and lysed by RLT buffer (Qiagen, Tokyo, Japan) supplemented with 2-mercaptoethanol. RNA was extracted using Agencourt RNAClean XP (Beckman Coulter, Tokyo, Japan) and reverse transcribed to cDNA with SMART-Seq v4 Ultra Low Input RNA Kit for Sequencing (Takara Bio, Tokyo, Japan). Sequence libraries were prepared with KAPA Hyper Prep Kit (KAPA Biosystems, Wilmington, MA), and 2×100 paired-end sequencing was performed with a HiSeq. 4000 system (Illumina, Tokyo, Japan).

### Transcriptomic analysis

Adaptor sequences were removed by Cutadapt^38^, and trimmed reads were mapped to human genome Grch38/hg38^39,40^ with HISAT2 version 2.0^41^. Read alignments were sorted and converted by SAMtools version 1.3.1^42^. The reads were normalized to fragments per kilobase of transcript per million mapped reads by Cufflinks version 2.2.1^43^. For identification of differentially expressed genes, PCA, and normalization of reads, raw tag counts were generated by featureCounts^44^ and processed using the DESeq. 2 package in R^45^. Gene expression was visualized by heat mapping with the ComplexHeatmap package in R^46^. Gene Ontology (GO) enrichment analysis was performed by calculating overlaps with biological process gene sets in MSigDB^47^. GSEA^48^ was performed with ranked gene lists based on q-value. TCR clones were reconstructed from the reads obtained by the above RNA sequencing using MiXCR^49^. Estimation of TCR repertoire diversity was performed with the tcR^50^ and VDJ tools^51^.

### Multi-cytokine production assay

TILs were suspended in AIM-V (Thermo Fisher Scientific, Tokyo, Japan) supplemented with 10% AB serum (Biowest, Nuaillé, France) and then treated with Golgi STOP (1:1000), PMA (50 ng/mL), and ionomycin (1 µM) for 4 h. Harvested cells were stained with Zombie Yellow Fixable Viability kit (BioLegend), anti-CD45-BV785, anti-CD45RA-APC-Cy7 [HI100], anti-PD-1-PE-Cy7, and anti-Tim3-PE. After fixing with Foxp3/transcription factor staining buffer set (Thermo Fisher Scientific), cells were stained with anti-CD3-BV421 [UCTH1], anti-CD4-APC [RPA-T4], CD8-BV711 [RPA-T8], anti-IL-2 [PerCP-eFluor710], anti-TNFα-BV510 [Mab11], and anti-IFNγ-FITC [4 S.B3].

### Cytotoxicity assay and intracellular staining

Cell surfaces were stained with Zombie Yellow Fixable Viability kit, anti-CD45-BV785, anti-CD45RA-APC-Cy7, anti-PD-1-PE-Cy7, and anti-Tim3-APC. Fixation and permeabilization of cells were conducted with Foxp3/Transcription Factor Staining Buffer Set (Thermo Fisher Scientific). CD3-Alexa Fluor 700, CD8-BV711, anti-Granzyme B-BV421 [GB11], and anti-perforin-FITC [B-D48] were used for intracellular staining.

### Patient follow-up

Patient follow-up generally included a history, physical examination, routine blood work, abdominopelvic computed tomography, and chest radiography. Details of the evaluation of therapeutic response were as previously reported^18^.

PFS time was retrospectively measured from the date of initiation of nivolumab treatment until disease progression, death from disease progression, or the date of the patient’s last follow-up visit. CSS time was retrospectively calculated from the date of initiation of nivolumab treatment until cancer-related death or the date of the patient’s last follow-up visit.

### Statistics

Comparisons between the surface marker expressions and clinical features were evaluated by Kruskal-Wallis test and Bonferroni-corrected Mann-Whitney U test. Distributions of PFS and CSS times were estimated with the Kaplan-Meier method, and associations between PFS, CSS, and the immunological classifications were assessed with a log-rank test. A value of *P* < 0.05 was considered statistically significant. These statistical analyses were performed with SPSS software, version 20.0 (IBM SPSS Statistics, Tokyo, Japan) and R (The R Foundation for Statistical Computing, Vienna, Austria).

## Supplementary information

Supplementary Fig. 1-6 and Table 1-2.
