# Supplementary material for: Tumour grade significantly correlates with total dysfunction of tumour tissue-infiltrating lymphocytes in renal cell carcinoma
Source: Sci Rep. 2020 Apr 10;10:6220. doi: 10.1038/s41598-020-63060-1 (PMC7148296; doi:10.1038/s41598-020-63060-1)
Supplement: Supplementary file 1 — Supplementary Fig. 1-6 and Table 1-2. [file 41598_2020_63060_MOESM1_ESM.pdf]

Tumour grade significantly correlates with total dysfunction of tumour tissue-infiltrating lymphocytes in renal cell carcinoma

Atsunari Kawashima[1†,*], Takayuki Kanazawa[2,3†], Yujiro Kidani[3,4], Tetsuya Yoshida[3,4], Michinari Hirata[2,3], Kentaro Nishida[2], Satoshi Nojima[5], Yoshiyuki Yamamoto[1], Taigo Kato[1,5], Koji Hatano[1], Takeshi Ujike[1], Akira Nagahara[1], Kazutoshi Fujita[1], Akiko Morimoto-Okazawa[2], Kota Iwahori[2], Motohide Uemura[1,6], Ryoichi Imamura[1], Naganari Ohkura[4], Eiichi Morii[5], Shimon Sakaguchi[7], Hisashi Wada[2], and Norio Nonomura[1]

[1]Department of Urology, [2]Department of Clinical Research in Tumour Immunology, Graduate School of Medicine, Osaka University, Suita, Osaka, Japan

[3]Drug Discovery & Disease Research Laboratory, Shionogi & Co., Ltd., Toyonaka, Japan

[4]Department of Basic Research in Tumour Immunology, [5]Department of Pathology, [6]Department of Urological Immuno-Oncology, [7]Department of Experimental Immunology, Immunology Frontier Research Centre, Graduate School of Medicine, Osaka University, Suita, Osaka, Japan

Supplementary Figure legends

Supplementary Figure 1. Gating and data acquisition of flow cytometry of 3 samples analysed in RNA sequencing. Expression of CD103 were evaluated within each fraction of CD8+ T cells.

Supplementary Figure 2. Rarefaction analysis of T-cell receptor (TCR) repertoire samples from 4 subsets of Fr. I, Fr. II, Fr. III, and Fr. IV of CD8+ T cells of 3 patients (Pt1–Pt3) (Left: TCRA, Right: TCRB). Solid and dashed lines mark interpolated and extrapolated regions of rarefaction curves, respectively, and points mark exact sample size and diversity. Shaded areas mark 95% confidence intervals (**a**). Comparison of TCRA and TCRB repertoire diversities using normalized Chao1 estimate (**b**). Clonal space homeostasis of TCRA and TCRB within each fraction of 3 patients (**c**). Venn diagrams representing TCRA (upper) and TCRB (lower) repertoire clones among 4 subsets for each patient (**d**). Statistical analysis for multiple comparisons was performed by Kruskal-Wallis analysis. The central tendency of the box plot indicates the median of each group, and the upper and lower ranges of the box plot show the 25th and 75th percentiles of each data set, respectively.

Supplementary Figure 3. Rarefaction analysis of T-cell receptor (TCR) repertoire samples from 4 subsets of Fr. I, Fr. II, Fr. III, and Fr. V of CD4+ T cells of 3 patients (Pt1–Pt3) (Left: TCRA, Right: TCRB). Solid and dashed lines mark interpolated and extrapolated regions of rarefaction curves, respectively, and points mark exact sample size and diversity. Shaded areas mark 95% confidence intervals (**a**). Comparison of TCRA and TCRB repertoire diversities using normalized Chao1 estimate (**b**). Clonal space homeostasis of TCRA and TCRB within each fraction of 3 patients (**c**). Venn diagrams representing TCRA (upper) and TCRB (lower) repertoire clones among 4 subsets for each patient (**d**). Statistical analysis for multiple comparisons was performed by Kruskal-Wallis analysis. The central tendency of the box plot indicates the median of each group, and the upper and lower ranges of the box plot show the 25th and 75th percentiles of each data set, respectively.

Supplementary Figure 4. Comparison of the multiple cytokine productivity (IFNγ, TNFα, and IL-2) of CD4 TILs from 12 patients with both high CD4 Fr. V and CD8 Fr. IV stratified by tumour grade within Fr. II, Fr. III, and Fr. V. Comparison of two sample types was performed by Mann-Whitney U test. The central tendency of the box plot indicates the median of each group, and the upper and lower ranges of the box plot

show the 25th and 75th percentiles of each data set, respectively. $^*P < 0.05$.

Supplementary Figure 5. (a) Table indicating the expression of CD8 Fr. IV and CD4 Fr. V T cells and clinical and pathological items within 10 patients treated with nivolumab monotherapy among 97 patients analysed **(b)**Probability estimates of cancer-specific survival time of the above 10 patients and stratification by expression of dysfunctional populations (upper left), combination of tumour grade and expression of dysfunctional populations (upper right), and tumour grade (lower). Statistical analysis was performed by log-rank test.

Supplementary Figure 6. Flowchart illustrating patient cohorts that were evaluated for this analysis.

Supplementary Fig. 1

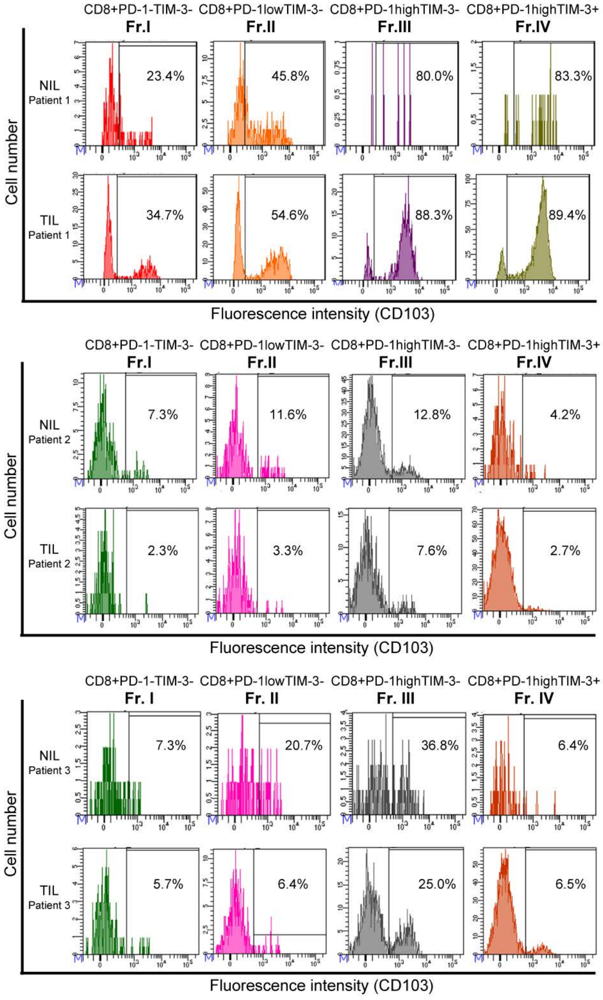

Supplementary Fig. 2

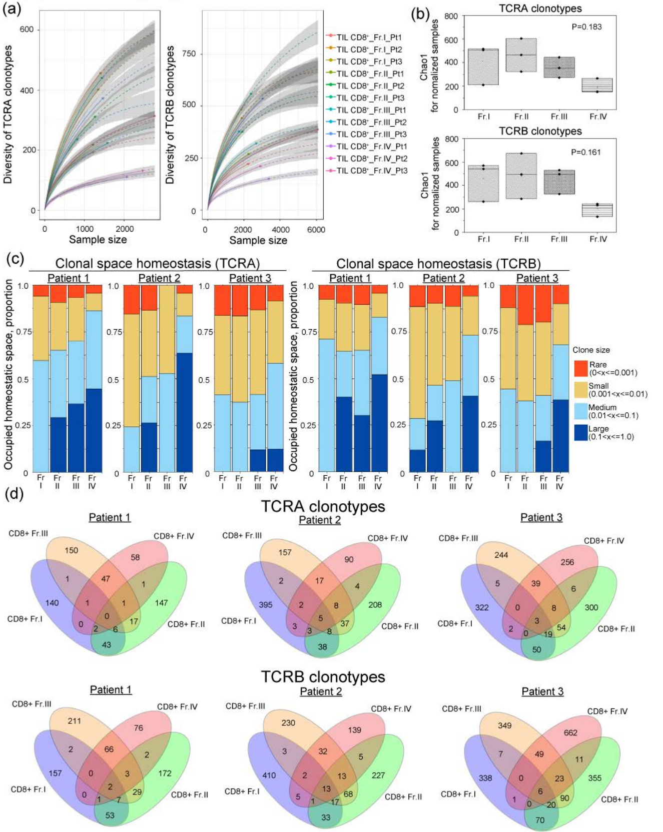

Supplementary Fig. 3

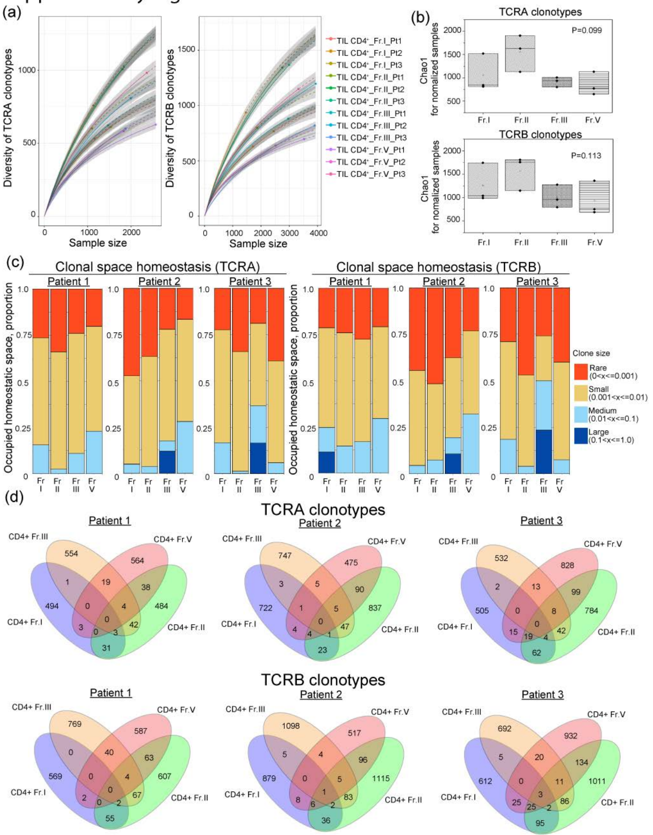

Supplementary Fig. 4

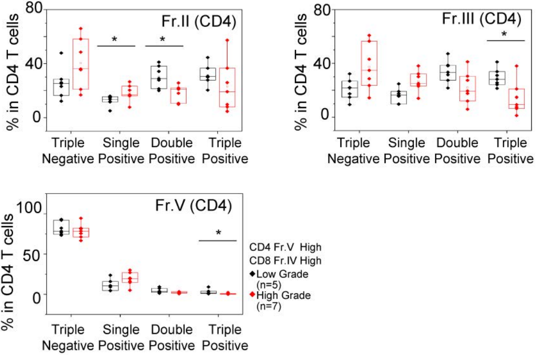

Supplementary Fig. 5

(a)

Sample ID	Age	Gender	WHO/ISUP grade	WHO/ISUP grade	CD8 Fr. IV /CD8 (%)	CD8 Fr.IV	CD4 Fr.V /CD4 (%)	CD4 Fr.V	Coagulative Necrosis
URO42	63	Male	3	High	3.99	Low	8.99	High	Presence
URO115	75	Male	2	Low	3.50	Low	8.30	High	Presence
URO195	60	Male	4	High	68.27	High	10.54	High	Presence
URO3	80	Male	2	Low	16.88	Low	2.13	Low	Presence
URO223	65	Male	4	High	65.0	High	18.0	High	Absence
URO86	67	Female	4	High	50.73	High	4.66	High	Absence
URO174	69	Female	2	Low	24.89	High	11.87	High	Absence
URO181	67	Female	3	High	60.01	High	9.15	High	Presence
URO204	66	Male	3	High	2.52	Low	3.76	High	Presence
URO76	72	Female	2	Low	5.33	Low	0.12	Low	Presence

(b)

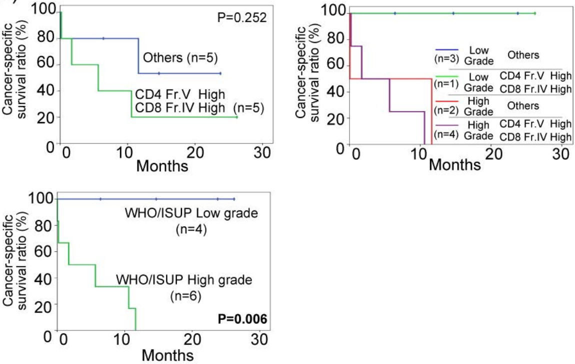

Supplementary Fig. 6

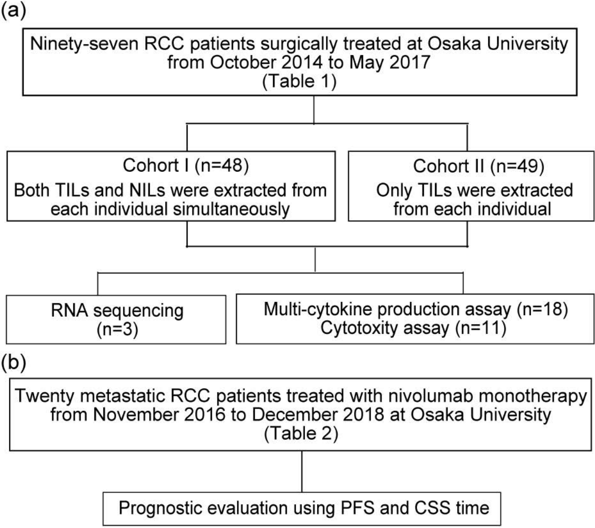

Supplementary Table 1. Clinical characteristics of the patients (n = 97)	
Age (median) (years)	27–82 (66)
Gender	
Male	70
Female	27
Histological type	
Clear cell	86
Clear cell with sarcomatoid change	1
Sarcomatoid	1
Papillary	4
Chromophobe	2
Mucinous tubular and spindle	1
Unclassified	2
pT stage	
1a/1b	43 / 15
2	6
3a/3b/3c	20 / 10 / 3
WHO/ISUP grade	
1 / 2 / 3 / 4	5 / 63 / 20 / 9
Coagulative necrosis	
Absence	72
Presence	25
INF	
A	75
B	22
pN stage	
0	92
I	5
M stage	
0	86
I	11
Lymphovascular invasion	
Yes	31
No	66
NLR (mean)	0.82 – 9.68 (2.71)
PLR (mean)	0.26 – 4.65 (1.05)
CRP (median) (mg/dl)	0.0 – 18.34 (0.09)
Follow-up time (median) (months)	0.49 – 44.19 (23.93)

Abbreviations: INF = infiltration; NLR = neutrophil to lymphocyte ratio; PLR = platelet to lymphocyte ratio; CRP = C-reactive protein.

Supplementary Table 2. Clinical characteristics of the 20 patients treated by Nivolumab	
Age (median) (years)	45 – 81(66)
Gender	
Male	15
Female	5
Histological type	
Clear cell	17
Papillary	1
Unclassified	2
WHO/ISUP grade	
1	1
2	8
3	6
4	5
Coagulative necrosis	
Absence	10
Presence	10
MSKCC risk classification	
Favourable	2
Intermediate	11
Poor	7
Number of prior therapies	
1	7
2	7
3	3
4	0
5	1
6	1
7	1
NLR (median)	1.44 – 6.90 (3.47)
PLR (median)	0.15 – 3.76 (1.17)
CRP (median) (mg/dl)	0.04 – 17.12 (1.11)
Follow-up time (median) (months)	0.03 – 30.87 (10.6)

Abbreviations: MSKCC = Memorial Sloan Kettering Cancer Center; NLR = neutrophil to lymphocyte ratio; PLR = platelet to lymphocyte ratio; CRP = C-reactive protein.
